# Efficient Cocaine Degradation by Cocaine Esterase-Loaded Red Blood Cells

**DOI:** 10.3389/fphys.2020.573492

**Published:** 2020-09-10

**Authors:** Luigia Rossi, Francesca Pierigè, Marco Agostini, Noemi Bigini, Veronica Termopoli, Yingting Cai, Fang Zheng, Chang-Guo Zhan, Donald W. Landry, Mauro Magnani

**Affiliations:** ^1^Department of Biomolecular Sciences, University of Urbino, Urbino, Italy; ^2^EryDel S.p.A., Milan, Italy; ^3^Laboratory of Toxicology, ASUR AV1, Pesaro, Italy; ^4^Department of Pure and Applied Sciences, University of Urbino, Urbino, Italy; ^5^Molecular Modeling and Biopharmaceutical Center, College of Pharmacy, University of Kentucky, Lexington, KY, United States; ^6^Department of Pharmaceutical Sciences, College of Pharmacy, University of Kentucky, Lexington, KY, United States; ^7^Department of Medicine, Columbia University, New York, NY, United States

**Keywords:** cocaine use disorder, cocaine esterase, red blood cells as drug delivery system, cocaine degradation, RBP 8000 stability, E196-301 stability

## Abstract

Recombinant bacterial cocaine esterase (CocE) represents a potential protein therapeutic for cocaine use disorder treatment. Unfortunately, the native enzyme was highly unstable and the corresponding mutagenized derivatives, RBP-8000 and E196-301, although improving *in vitro* thermo-stability and *in vivo* half-life, were a partial solution to the problem. For cocaine use disorder treatment, an efficient cocaine-metabolizing enzyme with a longer residence time in circulation would be needed. We investigated *in vitro* the possibility of developing red blood cells (RBCs) loaded with RBP-8000 and E196-301 as a biocompatible system to metabolize cocaine for a longer period of time. RBP 8000 stability within human RBCs is limited (approximately 50% residual activity after 1 h at 37°C) and not different as for the free enzyme, while both free and encapsulated E196-301 showed a greater thermo-stability. By reducing cellular glutathione content during the loading procedure, in order to preserve the disulfide bonds opportunely created to stabilize the enzyme dimer structure, it was possible to produce an encapsulated protein maintaining 100% stability at least after 4 h at 37°C. Moreover, E196-301-loaded RBCs were efficiently able to degrade cocaine in a time- and concentration-dependent manner. The same stability results were obtained when murine RBCs were used paving the way to preclinical investigations. Thus, our *in vitro* data show that E196-301-loaded RBCs could act as efficient bioreactors in degrading cocaine to non-toxic metabolites to be possibly considered in substance-use disorder treatments. This approach should now be investigated in a preclinical model of cocaine use disorder to evaluate if further protein modifications are needed to further improve long term enzyme stability.

## Introduction

It was recently reported that cocaine was the most commonly used illicit stimulant drug in Europe with lifetime use of 12.4 million males and 5.7 million females (European Monitoring Centre for Drugs and Drug Addiction, 2019) and that benzodiazepines, cocaine, or methamphetamine were present in 63% of opioid deaths during January–June 2018, among 13,631 opioid deaths in the 25 states (The United States Centers for Disease Control and Prevention on August 30, 2019; Gladden et al., 2019). The situation is also critical in many other areas including South America. The Unodc World Drug Report (2019) estimated that global illicit manufacture of cocaine reached an all-time high of 1,976 tons in 2017, with a 25% increase on the previous year. Substance abuse is also a significant economic cost to society in terms of healthcare expenses, loss of workplace productivity, justice costs, etc. Thus, cocaine abuse is a serious problem in many countries with neither a solution nor an approved treatment (Karila et al., 2008; Xi and Gardner, 2008). Addiction recovery programs are the main treatment options (Source: National Institute on Drug Abuse; National Institutes of Health; United States Department of Health and Human Services).

As described by Schulz and Schmoldt (2003), cocaine concentration in the blood of addicts is in the 0.5–1.0 μg/ml range (*t*_1/2_ of approximately 0.5–1.0 h) while a comatose-fatal dose is from 4 μg/ml. In humans, approximately 40% of cocaine is hydrolyzed to ecgonine methyl ester, a biologically inactive metabolite, by plasma enzyme butyrylcholinesterase (BChE) and liver carboxyesterase-2; moreover, more cocaine is biotransformed to benzoylecgonine via hydrolysis catalyzed by carboxyesterase-1, and to norcocaine via oxidization catalyzed by liver microsomal cytochrome P450 (CYP) 3A4 (Zheng and Zhan, 2012; Holmes, 2015).

Several potential therapeutics have been tested and/or are under development, including immunotherapy (by a specific vaccine or specific monoclonal antibodies; Carrera et al., 2004), N-acetylcysteine (Deepmala et al., 2015), Modafinil (Kampman, 2019), and several others (Forray and Sofuoglu, 2014).

Unfortunately, drawbacks exist for most of them: engineered bacteriophages, displaying cocaine-binding antibodies on their surface, intranasally administered as vaccine to sequester cocaine in the brain, could enter the periphery thus activating the immune system; N-acetylcysteine, also if showing in a phase I trial encouraging results in reducing cocaine related withdrawal symptoms and craving (LaRowe et al., 2006), it was not able to modulate basal glutamate concentrations in the nucleus accumbens of cocaine-addicted individuals (Engeli et al., 2020); no evidence of efficacy of Modafinil in increasing cocaine abstinence and treatment retention rate was observed in clinical studies (Sangroula et al., 2017). Other pharmacological strategies for cocaine use disorder treatment thus far include the use of glutamatergic and GABAergic agents such as topiramate or combinations of topiramate and long-acting amphetamine. There have been several positive trials of topiramate for cocaine use disorder, although there have been several trials that yield negative results as well (Johnson et al., 2013; Kampman et al., 2013; Nuijten et al., 2014). In addition, Topiramate also has side effects that may make it difficult to tolerate fatigue and general mental slowing. Among the most promising adjunct therapy for the treatment of cocaine dependence are: (a) Cannabidiol due to its effect on cocaine consumption, even if the evidence is limited to animal models and further research and clinical trials are needed (Rodrigues et al., 2020); (b) Doxazosin due to its ability in reducing cocaine use in individuals with the *ADRA1D* gene polymorphism (T1848A; Shorter et al., 2020); (c) Lorcaserin, 5-HT2CR agonist, to reduce cocaine self-administration (Phase 1 clinical trial, NCT02537873); (d) CI-581a and CI-581b, glutamate modulators tested in conjunction with motivational enhancement therapy (MET), to promote and maintain individuals’ abstinence from cocaine (Phase III clinical trial, NCT03344419).

Despite many efforts, no medications have yet been proven to be safe and effective for the treatment of cocaine use disorder.

Of relevance, the use of the recombinant bacterial cocaine esterase (CocE;Narasimhan et al., 2012) was demonstrated *in vitro* and *in vivo* to represent a potential therapeutic protein that can be used for the degradation of cocaine to non-toxic metabolites. Unfortunately, the native enzyme was highly unstable (half-life about 12 min at 37°C) and the corresponding mutagenized derivative, although improved *in vitro* thermo stability up to 6 h at 37°C and *in vivo* half-life (Gao et al., 2009), was not a solution to the problem. In fact, the reported thermostable mutant of CocE (known as drug RBP-8000) designed through computational modeling and simulations (Gao et al., 2009), has been advanced to placebo controlled clinical trial phase II for cocaine overdose treatment (Nasser et al., 2014). The results obtained provide strong evidence supporting the use of CocE as a pharmacotherapy for cocaine overdose treatment. However, for cocaine use disorder treatment, a highly efficient cocaine-metabolizing enzyme with a residence time in circulation as long as possible would be needed. More recently, Fang et al. (2014) designed and produced a new CocE mutant (denoted E196-301) with additional mutations to stabilize the dimer structure of the enzyme by cross-subunit disulfide bonds. E196-301 showed an *in vitro* half-life > 100 days and protected mice from a lethal dose of cocaine of 3 days. This is a great advancement with respect to the state of the art in this field but should likely be further improved in order to generate a cocaine abuse treatment.

Although several methods are available to extend *in vivo* half-life of therapeutic proteins, none surpasses the use of red blood cells (RBCs) as carriers for therapeutic enzymes (Leuzzi et al., 2016; Rossi et al., 2016). In fact, human RBCs circulate for about 120 days, are removed from the blood stream only when they are recognized as senescent and not by random processes and, most importantly, can be loaded *ex vivo* with therapeutic proteins without damaging the loaded cells that continue to have a normal circulation *in vivo* (Coker et al., 2018). Based on these premises, we have investigated the possibility to load RBCs with both CocEs: T172R/G173Q mutagenized protein (already designed RBP 8000) and the new E196-301 enzyme that, in addition to the T172R/G173Q modifications, also contains the L196C/I301C mutations. The results reported in this paper suggest that the stability within human RBCs of RBP 8000 is limited and not different from its free enzyme; on the other hand, the performances of E196-301-loaded RBCs are significantly better and *in vitro* they are very efficient in degrading cocaine to non-toxic metabolites. However, the E196-301 stability is influenced by reduced glutathione (GSH) concentration inside the loaded cells, limiting the applicability of the engineered cellular bioreactor. Further enzyme stabilizations that are not redox-dependent will likely provide the final solution to this problem.

## Materials and Methods

### Mutagenized CocE

T172R/G173Q mutagenized CocE (RBP 8000), dissolved in 1% sodium phosphate buffer and showing increased stability compared to the wild type enzyme, was provided by Columbia University (United States). Protein concentration was 10 mg/ml (by Bradford assay) and specific activity (SA) was estimated by the authors to be 112 U/mg.

**FIGURE 1:**
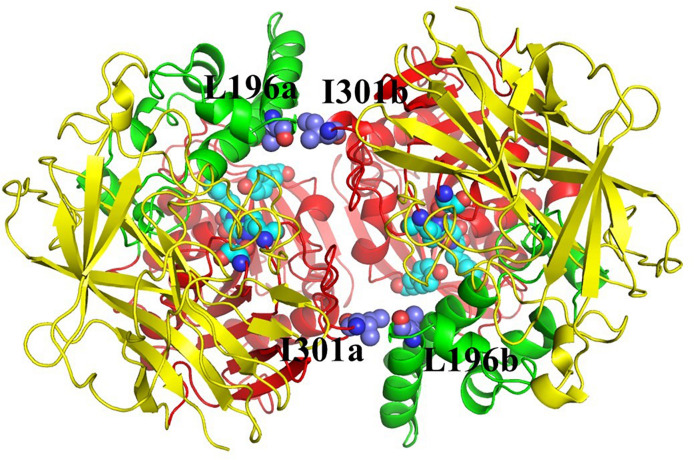
Crystal structure of the cocaine esterase (CocE) dimer. Ribbon representation of the homodimeric molecule showing the side chains of the soleucine and leucine residues that will be replaced with cysteine forming intersubunit disulfide bonds.

Cocaine esterase E196-301, containing the L196C/I301C mutations in addition to the T172R/G173Q modifications present in the RBP 8000, was provided by University of Kentucky (United States). The amino acids leucine and isoleucine, replaced with cysteine, are shown in Figure 1. These mutations produce a pair of cross-subunit disulfide bonds (on the dimer interface) that stabilize the dimer structure: C196a-C301b and C301a-C196b. C196a-C301b forms a disulfide bond between C196 of subunit a (C196a) and C301 of subunit b (C301b) while C301a-C196b forms a disulfide bond between C301 of subunit a (C301a) and C196 of subunit b (C196b). Protein concentration was 10 mg/ml (by Bradford assay) and SA is estimated by the authors to be 150 U/mg.

### Enzymatic Assay to Evaluate CocE Activity

Cocaine esterase catalyzes the hydrolysis of cocaine to ecgonine methyl ester and benzoate. For convenience, CocE activity has been evaluated with the alternative substrate butyrylthiocholine (BTC) since it was not hydrolyzed by the RBC acetylcholine esterase. CocE catalyzes the hydrolysis of BTC to thiocholine and butyrate; the rate of production of thiocholine is measured by following the reaction between thiocholine and 5,5’-dithiobis-(2-nitrobenzoic acid) (DTNB) at 412 nm (Beutler, 1984a).

### Determination of GSH in RBCs

Intracellular GSH concentration was measured spectrophotometrically at 412 nm by Butler’s DTNB–based method (Beutler, 1984b).

### Loading of CocE in Human RBCs

Whole blood was obtained from healthy volunteers (20 ml total blood volume) included in the Italian blood donor registry [registered Associazione Volontari Italiani del Sangue (AVIS) donors] who signed an informed consent form. Blood, collected in heparinized tubes, was provided by the Santa Maria della Misericordia Hospital in Urbino. Briefly, CocE was loaded in human RBCs by means of hypotonic dialysis, isotonic resealing and re-annealing essentially as described in Magnani et al. (1988).

The dialysis procedure was carried out with different concentrations of protein added to RBCs suspended in Hepes buffer (154 mM NaCl, 10 mM Hepes, 5 mM glucose, pH 7.4) at different haematocrit (Ht) to a final volume of 1 ml, as follows:

A. 2 mg of RBP 8000 (200 μl protein solution [10 mg/ml] with SA 112 IU/mg) at 70% Ht.
B. 3 mg of RBP 8000 (300 μl protein solution [10 mg/ml] with SA 112 IU/mg) at 60% Ht.
C. 4 mg of RBP 8000 (400 μl protein solution [10 mg/ml] with SA 112 IU/mg at 50% Ht.
D. 3 mg of E196-301 (300 μl protein solution [10 mg/ml] with SA 150 IU/mg) at 60% Ht (dialysis buffer with 3 mM GSH, that is the standard buffer).
E. 3 mg of E196-301 (300 μl protein solution [10 mg/ml] with SA 150 IU/mg) at 60% Ht (dialysis buffer without GSH).
F. 4 mg of E196-301 (400 μl protein solution [10 mg/ml] with SA 150 IU/mg at 50% Ht (dialysis buffer without GSH).
G. 4 mg of E196-301 (400 μl protein solution [10 mg/ml] with SA 150 IU/mg at 50% Ht (dialysis buffer without GSH and with a pre-dialysis step, always without GSH).

The final osmolarity of the hypotonic solution was 70 ± 2 mOsm, measured by Osmometer Fiske Associates, Model 210 (Norwood, MA, United States). After dialysis, the cells reached about 92 ± 4 mOsm. To test if the removal of intracellular GSH was beneficial for the stability of enzyme, a condition (G) was performed by introducing a pre-dialysis step before adding the protein. When pre-dialysis step was performed, the human RBCs were dialyzed first alone (30 min), then with the protein replacing the same fresh amount of dialysis buffer. Unloaded RBCs, subjected to the same procedure without the addition of the recombinant proteins, were used as controls. The amount of entrapped enzyme was determined by the kinetic assay as reported earlier on an aliquot of final loaded RBCs at 10% Ht in phosphate-buffered saline solution (PBS) containing NaCl 137 mM, KCl 2.7 mM, Na_2_HPO_4_ 10 mM, and KH_2_PO_4_ 1.8 mM added with 5 mM glucose, successively diluted 1:20 in distilled water.

### Loading of CocE in Murine RBCs

Blood (about 1 ml) was collected from each anesthetized BTBR mouse (20 ml of total blood volume) by puncture of the retro-orbital sinus in heparinized tubes and murine RBCs were essentially processed as previously described for human RBCs: 1, 2, and 3 mg of enzyme were added to RBC suspensions at 60% Ht. First, murine RBCs were pre-dialyzed alone (30 min) and then each condition was dialyzed 1 h at 4°C vs. dialysis buffer optimized for murine RBC loading: 15 mM NaH_2_PO_4_, 15 mM NaHCO_3_, pH 7.4, 20 mM glucose, 4 mM MgCl_2_, and 2 mM ATP (75 mOsm). After dialysis the cells reached about 102 mOsm. Subsequent steps were then carried out as described for human cells and the amount of entrapped protein was determined. BTBR mice come from internal breeding and sacrificed for the maintenance of the colony. All experiments were conducted in accordance with European legislation (2010/63/UE), with Italian national legislation (DL26/2014) governing the use of animals for research and with the guidelines of the National Institutes of Health on the use and the care of laboratory animals (Authorization n. 486/2017-PR).

### *In vitro* Enzyme Stability

Stability of free and encapsulated CocE in human RBCs were evaluated *in vitro* in the presence of PBS added with 5 mM glucose for up to 20 h at 37°C. Enzyme-loaded RBCs were incubated at 10% Ht. At each time point (0, 1, 2, 3, 4, and 20 h), residual enzyme activity was measured spectrophotometrically as reported above (see section “Enzymatic Assay to Evaluate CocE Activity”). Stability of CocE encapsulated in murine RBCs was tested up to 4 h at 37°C. Results were reported as mean ± SD of three determinations performed in duplicate.

### *In vitro* Cocaine Degradation by Cocaine Esterase-Loaded RBCs

Cocaine (1 mg/ml in acetonitrile) and Cocaine-D3 (1 mg/ml in acetonitrile) were purchased from Sigma-Aldrich (Milano) and stored at -20°C until use. Authorization SP/116 of 16/10/2018, possession and scientific use of narcotic substances released to Prof. Achille Cappiello, Laboratory of Liquid Chromatography and Mass Spectrometry, University of Urbino.

The evaluation of cocaine degradation by CocE-loaded RBCs in the *in vitro* study was performed by incubating the erythrocytes at 37°C for up to 2 h in PBS pH 7.2 with 5 mM glucose containing 5 μg/ml cocaine to a final 40% Ht. This cocaine concentration, higher than that usually found in cocaine-dependent individuals, has been selected to appreciate the efficacy of the proposed bioreactor since at lower cocaine concentrations; CocE-loaded RBCs could have removed it too quickly. Cocaine extraction has been performed as follow: at planned time points (0, 2, 5, 10, 15, 30, 45, 60, 90, and 120 min) 50 μl RBC suspension in duplicate was lysed with an equal volume of distilled water and, after a cycle of freezing/thawing in dry ice, cold acetonitrile containing the internal standard (Cocaine-D3 0.1 ng/μl) was added, vortexed 1 minute for each sample, and centrifuged at 14,000 rpm at 4°C. The supernatants were immediately frozen. Cocaine detection has been performed by ultra performance liquid chromatography – tandem mass spectrometry (UHPLC-MS/MS) method internally validated in accordance with the ISS (Istituto Superiore di Sanità), Sector guidelines and ISO 17025.

### Statistical Analysis

Stability values of enzymatic activities of RBP8000 and E196-301 CocE, in free or encapsulated form, were analyzed by unpaired *t*-test (Mann–Whitney test). The effectiveness of the CocE-loaded RBCs to metabolize cocaine across time was analyzed by one-way ANOVA test.

## Results

### RBP 8000 Encapsulation in Human RBCs

Three different loading procedures, using blood samples from three different donors, were performed by varying at the same time both the RBC hematocrit and the concentration of enzyme during the dialysis step. It should be noticed that, to maximize RBP8000 loading into RBCs, by decreasing RBC Ht to 50%, a greater volume was available for free enzyme addition thus leading to a greater amount of the encapsulated protein (Table 1). Thus, RBP 8000-loaded human RBCs can act as cellular bioreactors endowed with defined amounts of CocE (see A–C in section “Loading of CocE in Human RBCs”).

**TABLE 1:** RBP 8000 encapsulation in human red blood cells (RBCs) under different experimental conditions.

**Loading**	**Samples**	**Free CocE mg (IU)**	**Dialysis Conditions**	**IU CocE/ml Packed RBCs**
I	a	4 (448)	50% Ht	165
	b	3 (336)	60% Ht	64
	c	2 (224)	70% Ht	24.5
II	d	4 (448)	50% Ht	180
	e	3 (336)	60% Ht	88
III	f	4 (448)	50% Ht	114
	g	3 (336)	60% Ht	45
**Mean ± SD**	a-d-f	4 (448)		153 ± 35
	b-e-g	3 (336)		66 ± 22

### *In vitro* Stability of RBP 8000 Cocaine Esterase Free and Encapsulated in Human RBCs

**FIGURE 2:**
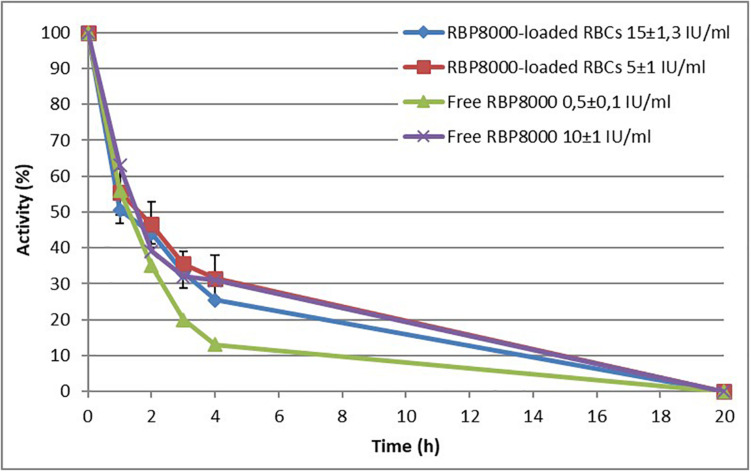
RBP 8000 stability. *In vitro* stability of free and red blood cell (RBC)-loaded RBP 8000 at 37°C in PBS, pH 7.2. Free enzyme was tested at 0.5 ± 0.1 and 10 ± 1 IU/ml. RBC-loaded enzyme was tested at 5 ± 1 and 15 ± 1.3 IU/ml RBCs. RBC suspensions at 10% Ht were used. All experiments were in duplicate that agree within 7.5%. Results are mean ± SD of three different experiments. Values of RBP 8000-loaded RBCs were not significantly different from control (free RBP 8000 at 10 ± 1 U/ml).

Stability of free RBP8000 was tested at 37°C in PBS containing 5 mM glucose at pH 7.2. Two different concentrations of the enzyme were used: 0.5 ± 0.1 and 10 ± 1 U/ml. As shown in Figure 2, residual enzyme activity after 1 h at 37°C ranged from 56 to 63% of the initial measured enzyme. The enzyme at the lowest concentration (0.5 U/ml) showed a fast decay at all-time points investigated. Thus, further studies were conducted excluding the lowest enzyme concentration. Human RBCs loaded with 5 ± 1 U/ml RBCs at 10% Ht and 15 ± 1.3 U/ml RBCs at 10% Ht were used. As reported in Figure 2, the RBP 8000 did not increase its stability within the RBCs with residual activity after 1 h at 37°C ranging from 50 to 55% of initial dose. Only about 30–35% residual activity was still present after 4 h at 37°C and at all-time points, the differences in stability of the free (10 ± 1 U/ml) vs. the RBC-loaded enzyme was not statistically different. Of note, no enzyme activity is detected after 20 h at 37°C in all conditions tested. In conclusion, the limited stability of RBP 8000, already reported (Gao et al., 2009), was confirmed and its encapsulation in human RBCs did not counteract its fast decay.

### *In vitro* Stability of E196-301 Cocaine Esterase Free and Encapsulated in Human RBCs

**FIGURE 3:**
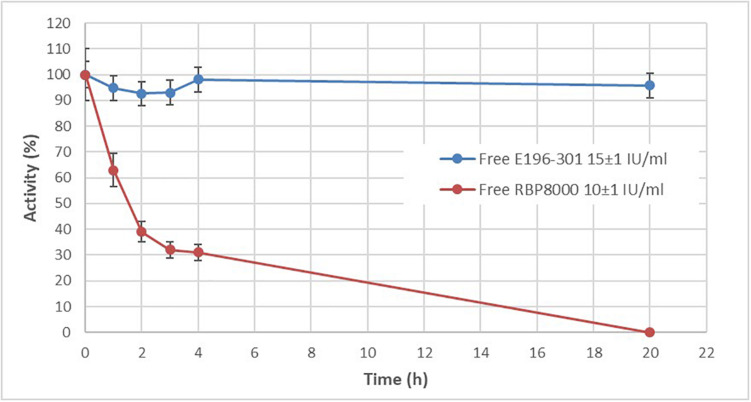
Stability comparison between RBP 8000 and E196-301. *In vitro* stability of RBP 8000 and 196-301 at 37°C in PBS, pH 7.2. Free enzymes were tested at 10 ± 1 and 15 ± 1 IU/ml, respectively. All experiments were in duplicate that agree within 7.5%. Results are mean ± SD of three different experiments. All values were significantly different (*p* < 0.005).

**FIGURE 4:**
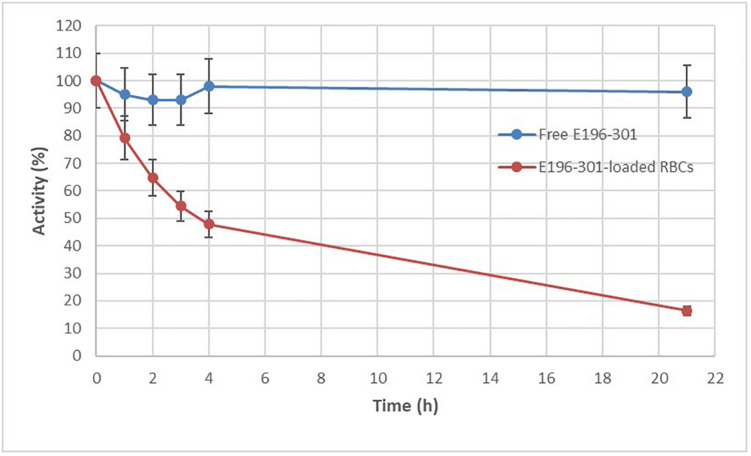
E196-301 stability in free and in red blood cell (RBC) encapsulated form. *In vitro* stability of free and RBC-loaded E196-301 at 37°C in PBS, pH 7.2. Free enzyme was tested at 15 ± 1 IU/ml, while RBC-loaded enzyme was tested at 16 ± 1 IU/ml RBCs. All experiments were in duplicate that agree within 7.5%. Results are mean ± SD of three different experiments.

The *in vitro* stability of RBP 8000 and E196-301 at 37°C, in PBS at pH 7.2 was next compared. As shown in Figure 3, E196-301 was significantly more stable than RBP 8000 under the investigated conditions. 96% residual activity is still measurable after 20 h at 37°C. The figure shows the mean of duplicate results of two experiments with almost identical values. The remarkable stability of E196-301 prompted to evaluate the enzyme stability within human RBCs. Encapsulation of E196-301 in human RBCs was very efficient. Starting from 3 mg of enzyme, human RBCs containing 90 ± 5 U/ml RBCs (see D in section “Loading of CocE in Human RBCs”), a value higher than that obtained using RBP 8000 under identical conditions (66 ± 22 U/ml RBCs), were developed. Unfortunately, when E196-301-loaded human RBCs were investigated for enzyme stability at 37°C, pH 7.2, the enzyme showed an unexpected instability (Figure 4). A residual activity accounting only for 48% of the initial units was observed already after 4 h; such percentage decreased to 18% after 20 h of incubation. Thus, E196-301 is much less stable in RBCs than in a physiological saline solution.

### Mechanisms of E196-301 Instability in Human RBCs

**FIGURE 5:**
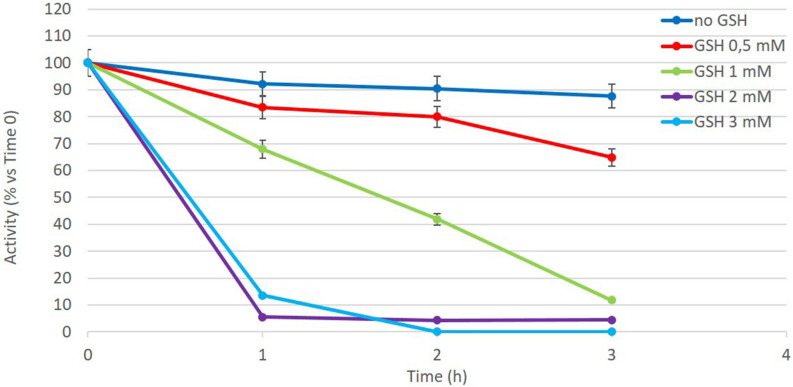
Reduced glutathione (GSH) effect on E196-301 stability. Enzyme activity has been evaluated at different times (0, 1, 2, and 3 h) after incubation at 37°C in a 1 ml-final volume in the absence or in the presence of different GSH concentration in the range 0.5–3 mM. Values are expressed in percentage respect to time 0. At this time the enzyme activity was 15 IU/ml. This is representative of two different duplicate experiments that agree within 5%.

The active enzyme is a dimer and possesses two disulfide bonds between subunits that vastly stabilize the enzyme, as established by structural studies by Fang et al. (2014). We reasoned that since the stability of E196-301 depends on the introduction of the two disulfide bonds, the reducing environment of the human RBCs could destabilize the enzymatic dimeric structure, thus causing a loss of stability. To verify this hypothesis, the stability of E196-301 at 37°C in the presence of increasing concentrations of GSH, the most abundant reducing agent in RBCs, has been investigated. As shown in Figure 5, E196-301 *in vitro* quickly loses its stability when GSH increases from 0.5 to 3 mM, a range of RBC physiological GSH concentrations.

### Encapsulation of E196-301 in Human RBCs Processed in the Absence and/or Presence of GSH

**FIGURE 6:**
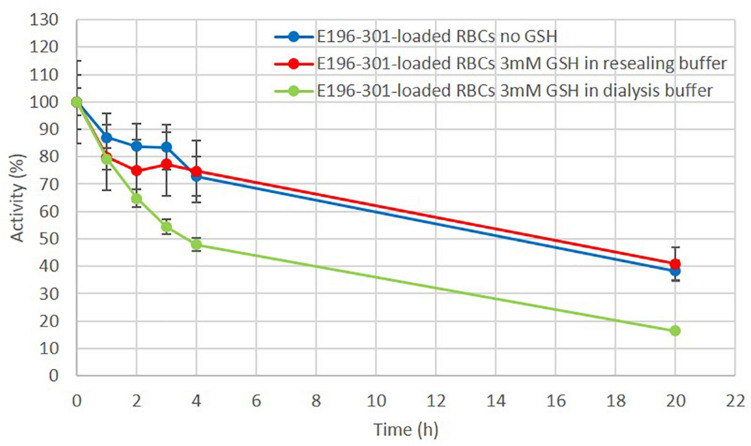
Effect of reduced glutathione (GSH) in E196-301 stability once encapsulated in red blood cells (RBCs). *In vitro* stability of RBC-loaded E196-301 at 37°C in PBS, pH 7.2. RBC-loaded enzyme was tested at 15 ± 2 IU/ml RBCs. All experiments were in duplicate that agree within 7.5%. Results are mean ± SD of three different experiments.

The enzyme loading experiments described above were performed in the presence of 3 mM GSH to preserve the reducing environment of the human erythrocytes during the encapsulation process. The removal of GSH from the buffer used to open the pores on the RBC membrane during the enzyme encapsulation process (see E in section “Loading of CocE in human RBCs”), was beneficial for the stability of E196-301 (Figure 6), with residual 75% activity after 4 h at 37°C and the addition of 3 mM GSH during the next resealing step did not affect this result. Thus, E196-301 in human RBCs is relatively stable if GSH is removed from the process solutions or added only at the final resealing step. At the same time, by performing a pre-dialysis step of RBCs alone before the addition of the protein to remove endogen GSH, an increase amount of active enzyme was observed (160 vs. 53 IU E196-301/ml packed RBCs, respectively; see G and F in section “Loading of CocE in human RBCs”). GSH concentration in native RBCs was 3.1 ± 0.06 mM (mean of three different blood samples in duplicate ± SD). After the enzyme encapsulation procedure, residual GSH ranged from 15 to 35% of this value (0.46 ± 0.03 to 1.1 ± 0.31) in six different blood samples submitted to the RBC encapsulation procedure carried on without the addition of the enzyme (sham RBCs).

### E196-301-Loaded Human RBCs Are Extremely Efficient in the Degradation of Cocaine

**FIGURE 7:**
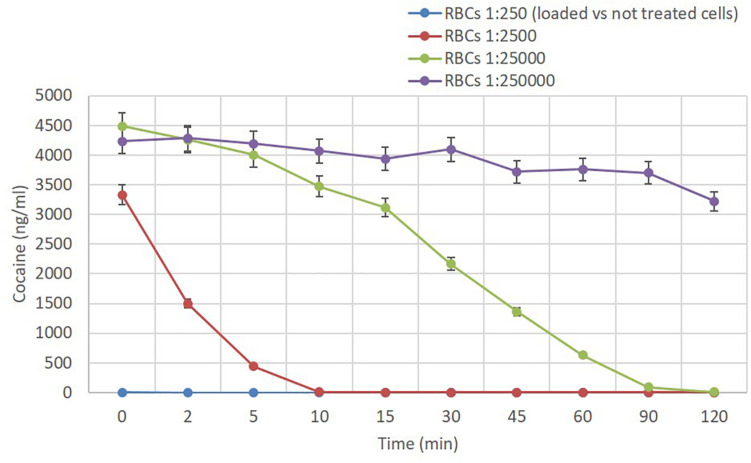
Cocaine degradation. The ability of E196-301-loaded red blood cells (RBCs) to metabolize cocaine has been evaluated at different times (range 0–2 h) after incubation at 37°C in a 500 μl-final volume with 5 μg/ml cocaine concentration. Conditions: 0.28 IU E196-301/ml RBC suspension (blue); 0.028 IU E196-301/ml RBC suspension (red); 0.0028 IU E196-301/ml RBC suspension (green); 0.00028 IU E196-301/ml RBC suspension (violet). This is representative of two different duplicate experiments that agree within 5%. For the RBC condition 1:2500 the times 0, 2, 5, and 10 min are highly significant among them (****); for the RBC 1:25000 condition each time is significantly different from the previous one up to time point 90 min (***); for the RBC condition 1:250000 a significant difference has been observed at each time (with the exception of the time points 2, 5, and 30 min) with respect to time zero (by one-way ANOVA test, ***p < 0.001, ****p < 0.0001).

The ability of E196-301 loaded RBCs to degrade extracellular cocaine was then investigated. Cocaine (500 ng/ml) at 37°C was found to be quite stable under the experimental conditions used. This low concentration was selected to appreciate also minimal cocaine degradation. After 3 h, more than 85% (range 84–86% in four experiments) of initial drug concentration was detectable. The presence of RBCs didn’t affect these data, suggesting that native RBCs don’t degrade cocaine. Instead, a suspension of human RBCs at 40% Ht loaded with 70 ± 5 U/ml of E196-301 (see G in section “Loading of CocE in human RBCs”) were extremely efficient in the degradation of extracellular cocaine. No residual cocaine was found also at the first time point of the experiment even with 5 μg/ml of cocaine present. Thus, the investigation has been repeated mixing E196-301 loaded human RBCs with native (non-processed) ones from the same donor. As shown in Figure 7 again, a dilution of enzyme-loaded RBCs in a ratio of 1:250 with native RBCs, removed immediately cocaine from the medium (PBS, pH 7.2, containing 5 mM glucose). Higher dilutions provided a proportional slower degradation of cocaine at 37°C that was still evident at 1:25,000 dilutions. During these experiments we have also evaluated the potential presence of extracellular E196-301 during the cocaine consumption studies and found to be negligible except when the CocE-loaded RBCs were investigated at 1:250 suspensions. In this case, extracellular E196-301 ranged from 1.3 to 2.0% (three experiments) of the encapsulated enzyme at the end of the experiment, excluding an eventual significant role in cocaine consumption.

### E196-301 Cocaine Esterase Can Be Efficiently Loaded Also in Murine RBCs

In order to carry out, in the future, *in vivo* studies on a preclinical model of cocaine use disorder, the possibility of producing E196-301-loaded murine RBCs was also evaluated. E196-301 was encapsulated in murine RBCs and its stability in physiological saline solution at 37°C, in comparison with the free form, was evaluated. The same loading procedure selected was performed by a RBC pre-dialysis step, without GSH in the dialysis buffer. Increasing amount of enzyme (1, 2, and 3 mg) has been added to RBC suspension at final hematocrit of 60% during the dialysis step. The results obtained are summarized in Table 2. As shown, adding increasing amounts of protein, RBCs loaded with increasing amounts of E196-301 have been obtained.

**TABLE 2:** Results of E196-301 encapsulation in optimized loading conditions.

**E196-301-Loaded RBCs 1 mg (IU/ml 100% Ht)**	**E196-301-Loaded RBCs 2 mg (IU/ml 100% Ht)**	**E196-301-Loaded RBCs 3 mg (IU/ml 100% Ht)**	* E196-301-Loaded RBCs Entrapment (%)	*Cell Recovery (%)
47	103	160	3	25

* The percentages of enzyme entrapment and cell recovery were the same for all the conditions tested (E196-301-L 1 mg, E196-301-L 2 mg and E196-301-L 3 mg). RBCs, red blood cells.

*In vitro* stability of free and encapsulated CocE has been evaluated by incubation in PBS with 5 mM glucose for 4 h at 37°C. Loaded murine RBC sample was investigated at 10% Ht (mean starting activity 5, 10, and 15 IU/ml) and free protein was tested at the concentration of 15 IU/ml. Data reported in Table 3 show that protein exhibits a high stability in the experimental settings: after 4 h-incubation CocE entrapped in murine RBCs kept its enzymatic activity intact.

**TABLE 3:** Stability of free or red blood cell (RBC) encapsulated E196-301.

**Time (h)**	**Free E196-301 Stability (%)**	**E196-301-Loaded RBCs 1 mg Stability (%)**	**E196-301-Loaded RBCs 2 mg Stability (%)**	**E196-301-Loaded RBCs 3 mg Stability (%)**
0	100	100	100	100
1	99	99	96	97
2	99	99	95	104
3	101	98	103	99
4	100	98	96	100

## Discussion

Cocaine abuse is a growing problem in many countries without an obvious solution, nor an approved treatment (Karila, 2008). Several potential therapeutics have been tested and/or are under development including therapeutic vaccines, antagonists, and many other approaches (Carrera et al., 2004; Xi et al., 2008; Forray and Sofuoglu, 2014; Deepmala et al., 2015; Kampman, 2019). Recently, the use of recombinant bacterial CoE (Narasimhan et al., 2012) has become a relevant possibility; in fact, CocE was demonstrated *in vitro* and *in vivo* to represent a potential therapeutic protein for the degradation of cocaine to non-addictive, non-toxic metabolites. However, the native enzyme showed a high instability (half-life about 12 min at 37°C); the improvement in thermostability by mutagenesis and the *in vivo* increased half-life (Gao et al., 2009) have represented only a partial solution to the problem. Indeed, although the use of CocE as a pharmacotherapy for cocaine overdose provided good results in clinical trials, a highly efficient cocaine-metabolizing enzyme with a longer residence time in circulation would be preferred for cocaine use disorder treatment. More recently, Fang et al. (2014) designed and produced a new CocE mutant (denoted as E196-301) with additional mutations that stabilize the dimer structure of the enzyme by cross-subunit disulfide bonds. E196-301 showed an *in vitro* half-life > 100 days and *in vivo* protected mice from a lethal dose of cocaine by 3 days when administered in the PEGylated form. Similar effects of PEGylation have also been reported for other mutant CocEs (Narasimhan et al., 2011; Collins et al., 2012). Indeed, PEGylation is a commonly used strategy to extend *in vivo* duration of action of therapeutics protein by reducing its immunogenicity and protecting it from the inactivation by anti-drug antibodies. However, many clinical studies have demonstrated that in some cases patients can also develop anti-PEG antibodies which caused adverse reactions and anaphylaxis (Hershfield et al., 2014; Gupta et al., 2018). Differently, by delivering the therapeutic enzymes through RBCs, PEGylation step can be avoided since the foreign enzyme is confined inside the erythrocytes, allowing it to be hidden from the immunological system and thus from any neutralizing antibodies that may be present in circulation. Thus, although several methods are available to extend *in vivo* half-life of therapeutic proteins, none surpasses the use of RBCs (Leuzzi et al., 2016; Rossi et al., 2016). In fact, human RBCs circulate for about 120 days and can be loaded *ex vivo* with therapeutic proteins without damaging the loaded RBCs (Coker et al., 2018). Based on these premises, we have investigated the possibility of loading human RBCs with both CocEs, namely the T172R/G173Q mutagenized protein (already designed RBP 8000) and the new E196-301 enzyme that, in addition to the T172R/G173Q modifications, also contains the L196C/I301C mutations. The results reported in this paper confirm that the stability within human RBCs of RBP 8000 is limited and not different from the free enzyme and that the performances of E196-301 loaded RBCs are significantly better and very efficient *in vitro* in degrading cocaine. Unfortunately, the E196-301 stability is influenced by GSH concentration inside the loaded cells possibly limiting the applicability of the engineered cellular bioreactor. GSH is the main reducing agent within RBCs and the E196-301 is stabilized by newly introduced cross-subunit disulfide bonds. The stability of the free enzyme *in vitro* is hampered by GSH and the loss of activity is concentration dependent within the physiological GSH concentrations present in RBCs. Furthermore, the maintenance of cellular GSH during the encapsulation procedure by the addition of external GSH during the loading step, promotes the loss of stability of the loaded CocE while the removal of GSH from the dialyzing solution, in addition to the GSH removal from RBCs by a pre-dialysis step, improves stability and activity recovery after the loading procedure. Through these improvements, the RBCs loaded with E196-301 are extremely effective in catalyzing the degradation of cocaine in a dose dependent manner, fulfilling all the requirements for a cell-based bioreactor for the treatment of cocaine use disorder.

As a proof of concept, our results suggest that a single infusion of 10 ml of a suspension of E196-301-loaded RBCs at 10% Ht (that is 1 ml packed RBCs), which exactly reproduce the dilution of enzyme-loaded RBCs in the ratio of 1:2500 with native RBCs shown in Figure 7, could be effective in quickly removing cocaine from circulation. Indeed, since plasma cocaine concentration in a dependent subject could reasonably be in the 10–1000 ng/ml range (Schulz and Schmoldt, 2003), corresponding to 0.165–16.5 μmol/5 L blood, the infusion of 160 IU of E196-301 (see Table 2) will be extremely efficient in a rapid and complete cocaine degradation. This conclusion is also supported by two recent studies in which cocaine has been administered intravenously or *per os* (Coe et al., 2018) or nasal insufflation (Menzies et al., 2019) and *C*_max_ values ranging from 45.1 to 925 ng/ml plasma and 347.5–517.7 ng/ml plasma, respectively, have been found. Moreover, Menzies et al. (2019) have also *in vivo* demonstrated that RBC cell wall presents no barrier to cocaine and its metabolites, thus corroborating our *in vitro* studies. Furthermore, since it is known that engineered RBCs produced for clinical use by the “Red Cell Loader” device have a T50 survival approximately of 40 days (Coker et al., 2018), and that the infusion of 160 IU of E196-301 by RBCs is higher than that it is needed to quickly remove all blood cocaine after a single dose intake, it is reasonable to assume that a single infusion can be efficient in blood cocaine degradation both in the case of repeated intakes over time and in the case of higher doses intakes. Thus, efficient cell bioreactors capable of catalyzing cocaine degradation can be produced by decreasing the intracellular GSH content during the loading procedure. *In vitro* studies confirm that these cells are stable (not shown). It is worth noting that human RBCs can restore their physiological level of GSH (Raftos et al., 2010). Thus, we expect a reduction of E196-301 activity during *in vivo* RBCs circulation. Repeated enzyme-loaded administrations at biweekly intervals can be explored, instead of monthly intervals, as actually occurs for RBC based therapies (Rossi et al., 2004; Castro et al., 2007; Chessa et al., 2014; U. S. National Library of Medicine, 2016). As a further alternative, enzyme stabilizations by non-redox-dependent cross-subunit bonds will likely provide the solution to this problem.

In summary, we have proved that CocE can be encapsulated in human and murine RBCs, offering the possibility of developing circulating cellular bioreactors capable of an efficient cocaine degradation. Due to the intrinsic ability of the RBCs to restore basal GSH concentration, this approach should now be investigated in *in vivo* models to define the timeframe of efficacy for the enzyme loaded RBCs and establish the frequencies of administration by proper pharmacokinetics studies. In case of selected applications that require a long-lasting effect as in the treatment of cocaine use disorder, the CocE could eventually be modified by introducing cross subunit bonds insensitive to the RBCs redox state. Due to the absolute medical needs and the absence of proper treatments to diminish cocaine use disorder, further investigations in this field are needed and welcomed. The preliminary data collected in this paper could represent the basis for these future studies.

## Data Availability Statement

All datasets generated in this study are included in the article/supplementary material.

## Ethics Statement

Ethical review and approval was not required for the study on human participants in accordance with the local legislation and institutional requirements. The patients/participants provided their written informed consent to participate in this study.

## Author Contributions

All authors have contributed substantially to the present work and have seen and approved the manuscript being submitted.

## Conflict of Interest

MM and LR hold shares in EryDel S.p.A., a company with interests in the technology of RBC-based drug delivery.

The remaining authors declare that the research was conducted in the absence of any commercial or financial relationships that could be construed as a potential conflict of interest.

## Abbreviations

BTC: butyrylthiocholine
Coc-E: cocaine esterase
GSH: reduced glutathione
Ht: haematocrit
RBCs: red blood cells.
